# Simultaneous Improvement and Genetic Dissection of Salt Tolerance of Rice (*Oryza sativa* L.) by Designed QTL Pyramiding

**DOI:** 10.3389/fpls.2017.01275

**Published:** 2017-07-20

**Authors:** Yunlong Pang, Kai Chen, Xiaoqian Wang, Wensheng Wang, Jianlong Xu, Jauhar Ali, Zhikang Li

**Affiliations:** ^1^Institute of Crop Sciences, National Key Facility for Crop Gene Resources and Genetic Improvement, Chinese Academy of Agricultural Sciences Beijing, China; ^2^International Rice Research Institute Metro Manila, Philippines; ^3^Agricultural Genomics Institute, Chinese Academy of Agricultural Sciences Shenzhen, China; ^4^Shenzhen Institute of Breeding and Innovation, Chinese Academy of Agricultural Sciences Shenzhen, China

**Keywords:** designed QTL pyramiding, green super rice, grain yield, salt tolerance, tGBS

## Abstract

Breeding of multi-stress tolerant rice varieties with higher grain yields is the best option to enhance the rice productivity of abiotic stresses prone areas. It also poses the greatest challenge to plant breeders to breed rice varieties for such stress prone conditions. Here, we carried out a designed QTL pyramiding experiment to develop high yielding “Green Super Rice” varieties with significantly improved tolerance to salt stress and grain yield. Using the F_4_ population derived from a cross between two selected introgression lines, we were able to develop six mostly homozygous promising high yielding lines with significantly improved salt tolerance and grain yield under optimal and/or saline conditions in 3 years. Simultaneous mapping using the same breeding population and tunable genotyping-by-sequencing technology, we identified three QTL affecting salt injury score and leaf chlorophyll content. By analyzing 32M SNP data of the grandparents and graphical genotypes of the parents, we discovered 87 positional candidate genes for salt tolerant QTL. According to their functional annotation, we inferred the most likely candidate genes. We demonstrated that designed QTL pyramiding is a powerful strategy for simultaneous improvement and genetic dissection of complex traits in rice.

## Introduction

Rice (*Oryza sativa* L.) is one of the most important food crops, but its production is adversely limited by various abiotic and biotic stresses. Among them, the saline soils are one of the most damaging stresses (Shrivastava and Kumar, 2015), especially in the coastal areas of South and South East Asia where are the main rice growing and consuming areas (Khush, 1997; Ali et al., 2006). Overall, rice plants are moderately sensitive to salt stress, but rice germplasm shows considerable variability for salt tolerance (ST) and its related traits (Gregorio et al., 2002). Thus, breeding high-yielding varieties tolerant to salinity and other abiotic stresses is the best option to enhance the rice productivity of saline-prone areas (Li and Xu, 2007). However, the complexity of ST genetically and physiologically makes it a challenging task to improve rice ST by the conventional breeding approach (Hoang et al., 2016).

Salt stress at the seedling stage is crucial as it affects rice crop establishment. Unfortunately, rice plants are vulnerable to salt stress at this stage (Lutts et al., 1995). High salts normally induce osmotic stress and ion toxicity to rice plants. Higher concentrations of salts in the solution or soil make it harder for roots to uptake water and, accumulation of large amounts of salts in plants and poor tolerance to the accumulated Na^+^ of leaf tissues result in toxicity (Munns and Tester, 2008). Both osmotic and ionic stresses could inhibit plant growth, reduce photosynthesis rate, increase the formation of reactive oxygen species, and cause leaf damage or even plant death (Rahman et al., 2016). ST of rice seedlings is inherently complex and involves multiple pathways including morphological, physiological, and biochemical processes (Li and Xu, 2007). Till date, through various genetic tools such as over-expression, knockdown, mutant, map-based cloning and transcript expression analysis, more than 200 ST related genes have been identified (Molla et al., 2015). These genes are responsible for different mechanisms such as ionic equilibrium, osmotic adjustment, transcription regulation and signaling pathway, etc. (Molla et al., 2015).

QTL mapping strategy has been extensively applied to dissect the genetic architecture of ST in rice. Numerous studies have been carried out to identify QTL affecting rice ST related traits at seedling stage such as salt injury score, fresh and dry weight of shoot and root, Na^+^ and K^+^ content of shoot and root, and chlorophyll content (Koyama et al., 2001; Lin et al., 2004; Ren et al., 2005; Lee et al., 2006; Zang et al., 2008; Sabouri et al., 2009; Thomson et al., 2010; Cheng et al., 2011; Tian et al., 2011; Wang et al., 2012; Zheng H. et al., 2015). However, the DNA markers used in these studies were usually low density RFLP and SSR markers; so that most identified QTL cover very large chromosomal intervals containing hundreds of genes, which makes it difficult to directly infer candidate genes. The fine-mapping approach or map-based cloning has to be followed, which is extremely troublesome and time-consuming. Thus, the reported QTL and their associated markers have limited applications to rice ST breeding. With the development of genotyping technology and reduction of costs, getting millions of SNP markers through genotyping by sequencing (GBS) and high density SNP chips is increasingly easy (Chen et al., 2014; He et al., 2014), which considerably facilitates QTL mapping studies and their application in rice breeding (Mammadov et al., 2012). Utilizing high density SNP markers, one could markedly reduce the chromosomal interval covered by identified QTL and predict candidate genes according to their functional annotation. Further, with the completion of 3K Rice Genome Project (3K RGP) (3K RGP, 2014), sequences and millions of SNP markers of 3,000 accessions are now available (Alexandrov et al., 2015; Zheng T.Q. et al., 2015). If the mapping population is derived from the crosses between accessions in 3K RGP, we can easily find candidate genes inside of the QTL interval between parents or grandparents.

The final goal for all the researchers on the genetic and molecular dissection of complex traits by QTL mapping and cloning is to apply the obtained genetic and molecular information to improve the breeding efficiency. Unfortunately, results from the theoretical research have not yet significantly changed the way rice breeders do their breeding because possible epistasis and QTL × environmental interactions (Luo et al., 2001; Li et al., 2003). Since 1998, we have been practicing an integrated molecular breeding strategy to develop genome-wide trait-specific introgression lines (ILs) for simultaneous improvement and genetic dissection of complex traits. It was then followed by designed QTL pyramiding (DQP) or molecular recurrent selection using the ILs and genetic information generated from the ILs (Li et al., 2005; Li and Zhang, 2013). It resulted in the development of several high-yielding and multi-stress resistant varieties (Li and Xu, 2007; Guan et al., 2010; Ali et al., 2017). However, a DQP experiment is typically used to improve target traits based on known genetic and phenotypic information of target attributes in parental ILs. It remains unclear if this approach is useful for improving different target traits that were not selected in the parental ILs.

In this study, we were trying to answer this question by developing high yielding green super rice (GSR) lines with significantly improved ST using an F_4_ population derived from a DQP cross between two drought tolerant (DT) ILs. Our specific objectives are to: (1) develop promising rice lines with improved ST as well as improved grain yield under saline and normal irrigated conditions; (2) identify QTL and candidate genes for ST related traits utilizing high density SNP markers of tunable GBS (tGBS) (Schnable et al., 2013) technology and sequencing/SNP data from 3K RGP^1^.

## Materials and Methods

### Materials

**FIGURE 1:**
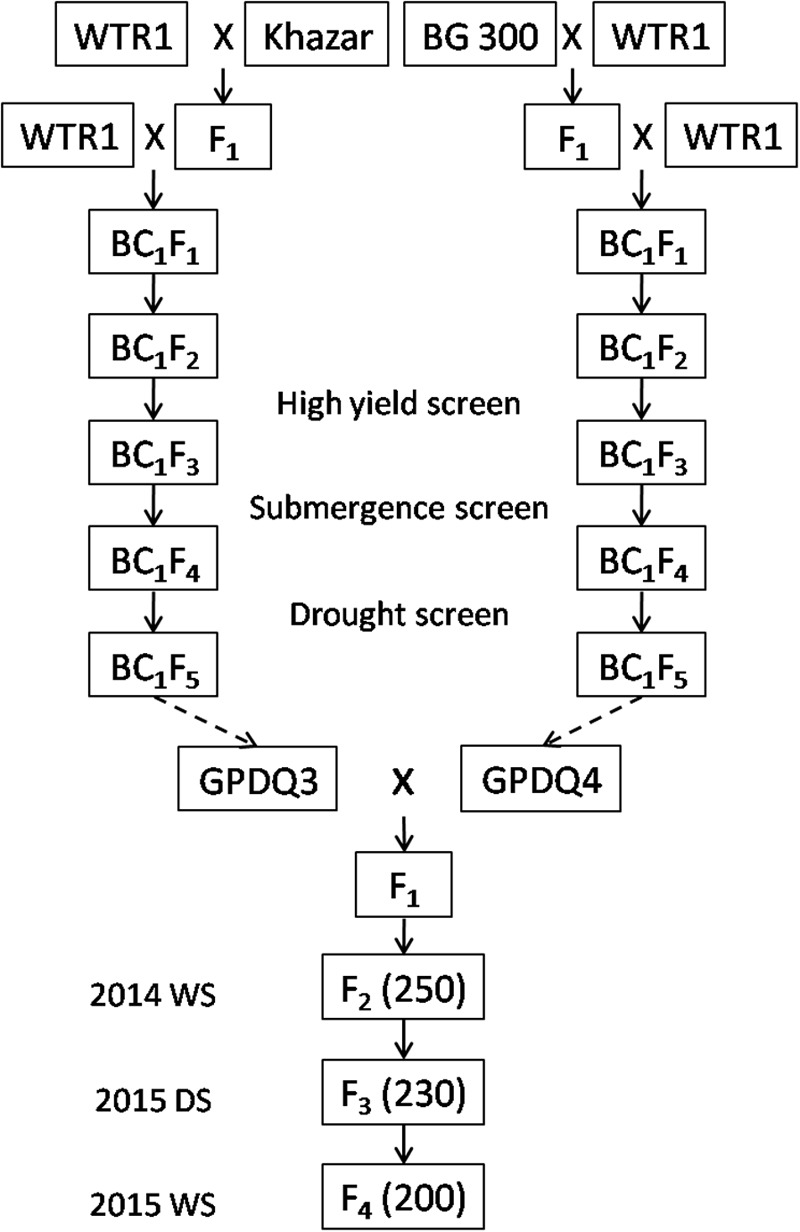
The process of developing 200 F_4_ lines by single seed decent from a pyramiding cross between two Weed Tolerance Rice 1 (WTR1) introgression lines. The values in brackets were the number of plants in each generation. WS and DS were wet season and dry season at International Rice Research Institute.

**Figure 1** shows the standard procedure for developing the breeding/mapping populations by DQP, which was demonstrated as a powerful breeding strategy for simultaneous improvement of yield potential and a single abiotic stress tolerance in which abiotic stress tolerance was the primary target trait (Guan et al., 2010; Li et al., 2017). In this study, we tried to test the possibility if DQP could be used for improving complex traits such as ST which was not the target trait in the original backcross breeding. Briefly, a DQP cross was made between two BC_1_F_5_ ILs, GPDQ3 and GPDQ4, in the genetic background of a high yield and widely adaptable rice variety, Weed Tolerance Rice 1 (WTR1) from South China. In the original backcross breeding effort, the donor parents of GPDQ3 and GPDQ4 are Khazar (aromatic type) from Iran and BG300 from Sri Lanka, respectively, and have moderate levels of ST. GPDQ3 and GPDQ4, were originally developed from the BC_1_F_2_ populations by going through substantial selections under drought, high yield and submergence conditions at International Rice Research Institute (IRRI). Thus, GPDQ3 and GPDQ4 had good levels of tolerance to drought and submergence as well as good yield potential. From the cross, 200 F_4_ lines developed by single seed descent method were used as the breeding/mapping population in this study.

### Methods

#### Phenotyping ST at the Seedling Stage

The phenotyping experiment was conducted in the greenhouse at IRRI, Los Baños, Philippines. The materials included 200 F_4_ lines derived from the cross between GPDQ3 and GPDQ4, and their grandparents, WTR1, Khazar, and BG300, plus the sensitive and tolerance checks, IR29 and FL478. A randomized complete block design was applied with three replications for each of the evaluated materials. Seeds of the testing materials were incubated for 5 days at 50°C to break dormancy. Sterilized seeds were then covered with moistened filter paper and incubated at 30°C for 48 h to germinate. One germinated seed was sown per hole on a Styrofoam seedling float with 100 holes in a plastic tray containing 8 L of Yoshida culture solution. At 14 days after sowing, NaCl was added to the culture solution to raise the electrical conductivity (EC) to 12 dS⋅m^-1^. The pH of the culture solution was adjusted daily to 5.0 by adding either NaOH or HCl (Yoshida et al., 1976) and the solution was renewed every 6 days, as described by Gregorio et al. (1997). Ten seeds of the checks (IR29 and FL478) were planted in each tray. The plants were scored based on visual symptoms of salt stress injury when the sensitive check IR29 was scored 7 as described in Standard Evaluation System (SES) for rice (IRRI, 2013). A SPAD-502 chlorophyll meter (Minolta Camera Co., Ltd., Japan) was used to measure leaf chlorophyll content of each seedling as the indicator of leaf senescence caused by salt stress. Measurements were made at the base, middle, and tip of the top leaves of each individual plant, and the average values were used in SPAD units.

#### Replicated Yield Trials

All salt selected pyramiding lines with SES from 1 to 3 were evaluated their grain yield performances under normal irrigated and saline conditions during the 2016 dry season (DS) and wet season (WS). Standard normal irrigated trial was conducted in the lowland rice field on the IRRI farm. The field was irrigated regularly and managed with standard crop management practices. Saline trial was conducted in a farmer’s field with natural coastal salinity located in Infanta, Quezon, Philippines. Salinity levels in the paddy were constantly monitored with an EM50 (Decagon Devices, Pullman, WA, United States) that ranged from 6.2 to 14.1 dS/m during the entire growth period from seeding to harvest, depending upon the rainfall and high tides. For both conditions, each line was planted in a three-row plot with eight individuals planted in each row at a spacing of 20 cm × 25 cm. A random complete block design was applied with two replications. At maturity, three uniform plants in the middle of each plot were bulk harvested, and the grain yield per plant (GY, in g) was measured.

#### Phenotypic Analysis

Phenotypic analysis was conducted using mixed linear models with lines (genotype) as the fixed effect and replications as the random effect. The best linear unbiased estimates (BLUE) of lines were obtained and were used in the following analysis. All analyses were conducted using the PBTools package of R (R Core Team, 2015) developed by IRRI^2^.

#### DNA Extraction and Genotyping

Genomic DNA was extracted from the seedlings of the 200 F_4_ lines, two parents (GPDQ3 and GPDQ4) and three grandparents (WTR1, Khazar, and BG300) using DNeasy Plant Mini Kit (Qiagen, United States) following the manufacturer’s protocol^3^. Then, these DNA samples were shipped to Data2Bio (Data2Bio 2079 Roy J. Carver Co-Lab 1111 WOI Road Ames, Iowa 50011-1085) for genotyping by tGBS technology (Schnable et al., 2013). The SNP calling was according to reference genome of Os-Nipponbare-Reference-IRGSP-1.0.

#### QTL Mapping

QTL mapping for salt injury score (measured by SES) and chlorophyll content was carried out using R/qtl package (Broman et al., 2003). Interval mapping (IM) was performed for both traits using the function ‘scanone’ to calculate the LOD value for each SNP. The LOD threshold was obtained based on permutation test (1000 permutations, *P* = 0.05) for each trait (Doerge and Churchill, 1996). The additive effect was calculated as the AA - (AA + BB)/2, where AA and BB is the mean phenotype value of genotype AA and BB, respectively. The phenotype variance explained (PVE) by each QTL was estimated by 1 - 10^-2LOD/n^, where n is the sample size and LOD is the LOD score (from ‘scanone’). The confidence interval of each QTL was delimited by the flanking markers within an 1 - LOD drop from the estimated QTL position. Significant QTL for each trait was named with the attribute, followed by numbers indicating the chromosome location and the numerical order of identified QTL on the chromosome. For example, *qChlo1.2* shows the second QTL for chlorophyll content on chromosome 1.

The selective genotyping method was also applied to detect QTL for ST. As the SES scores reflected the overall response of each line to the salt stress and was always used to identify ST plants (Rahman et al., 2016), here, we selected lines with SES ranging from 1 to 3 as the ST population. We firstly calculated the allele frequency in the selected ST population. Then, using the allele frequency in the whole random population as expected value, we tested the significance of segregation distortion of alleles in the selected ST population by chi-square tests. Finally, using the generated *p*-value, we calculated the *q*-value by the R package “*q*-value” (Dabney and Storey, 2004), and *q* = 0.05 was used as the threshold to determine significant segregation distortion SNPs.

#### Identification of Candidate Genes

For the identified QTL governing ST related traits, the genes located in their confidence interval were searched from The MSU Rice Genome Annotation Project Database^4^, and the genes having non-synonymous polymorphism loci between parents were regarded as candidate genes. As the genome sequences of parents (GPDQ3 and GPDQ4) were not available, we cannot identify the polymorphic genes in the identified QTL regions directly. Nevertheless, the grandparents (WTR1, Khazar, and BG300) were sequenced in 3K RGP and their 32M SNP genotype were available in the Rice SNP-Seek Database (Alexandrov et al., 2015). Therefore, we firstly searched the non-synonymous SNPs of genes in the grandparents and found the genes showing polymorphisms among them. Then we analyzed the graphical genotypes of GPDQ3 and GPDQ4 to determine the sources of chromosome fragments, which allowed us to identify different alleles of QTL from the parents.

## Results

### Phenotype Variance

Under the salt stress screening when sensitive check IR29 was scored 7, all the parents and grandparents were scored 5, and the SES scores of F_4_ lines ranged from 1 to 7. A total of 28 F_4_ lines showed the transgressive segregation with SES scores of 1–3 and were selected. BG300 and GPDQ4 had similar chlorophyll content of 32.5 and 29.9, respectively, which was higher than that of WTR1 (19.6), Khazar (21.5), and GPDQ3 (20.6). The chlorophyll content of F_4_ lines ranged from 2.6 to 42.2 with a mean of 22.1 (**Table 1**). The 28 selected lines with SES of 1–3 were progeny tested for their GY performances under the normal irrigated and salinity conditions in the 2016 DS and WS (**Table 2**). Under the normal irrigated condition, the parents, GPDQ4 and GPDQ3 had GY of 26.20 g (18.37 g) and 25.55 g (15.50 g) in the DS (WS), respectively. In the DS, the GY of the 28 selected lines ranged from 19.65 to 39.10 g with a mean of 28.33 g, and eight lines yielded significantly higher than that of GPDQ4 (the high value parent) with GY ranging from 31.60 to 39.10 g. In the WS, the GY of the 28 ST lines averaged at 17.94 g, and seven lines had significantly higher yield than that of the high value parent with GY advantages ranging from 9.8% to 21.9%. This led us to identify four promising lines D29G124, D29G111, D29G283, and D29G303 that showed significantly improved yield under the normal irrigated condition in both DS and WS. The genome-wide heterozygosity of the four promising lines ranged from 1.1% to 6.3%. Under the saline condition, GPDQ4 and GPDQ3 had GY of 13.17 g (14.30 g) and 11.92 g (10.07 g) in the DS (WS). In the DS, the GY of 28 selected lines ranged from 10.73 to 18.10 g with a mean of 12.91 g, with four lines showing significantly higher yield than GPDQ4 with yield advantages ranging from 13.7% to 37.4%. In the WS, the 28 selected lines had a mean GY of 13.29 g, with a range from 9.17 to 18.71 g per plant, and six lines had significantly higher yield than the high value parent with yield advantages ranging from 14.9% to 30.8%. This led us to identify three ST lines (D29G124, D29G105, and D29G301) that showed significantly improved yield potential under saline condition in both DS and WS. The genome-wide heterozygosity of the three ST lines ranged from 3.8% to 7.7%. Of the above six promising lines, D29G124 was the only one showing high yield under both the irrigated and saline conditions in the two consecutive seasons (**Table 2**).

**Table 1:** Phenotype variance of F_4_ lines, parents and grandparents.

Trait	Lines		Parents		Grandparents		
	Range	Mean ± SD	GPDQ3	GPDQ4	WTR1	Khazar	BG300
SES	1~7	4.4 ± 1.2	5	5	5	5	5
Chlorophyll content	2.6~42.2	22.1 ± 8.3	20.6	29.9	19.6	21.5	32.5

**Table 2:** The grain yield performances (g/plant) of 28 salt tolerant F_4_ lines under normal irrigated and saline conditions in 2016 dry season (DS) and wet season (WS) and their genomic heterozygosity.

tGBS.lD	SES	Irrigated		Saline		Heterozygosity
		DS	WS	DS	WS	
D29G124	1	39.10^∗^	22.40^∗^	14.97^∗^	17.19^∗^	0.0631
D29G159	1	23.55	19.17	12.60	13.34	0.0612
D29G160	1	32.30^∗^	18.13	12.93	11.88	0.0398
D29G111	2	34.85^∗^	20.79^∗^	18.10^∗^	10.85	0.0105
D29G123	2	22.50	18.98	13.60	18.37^∗^	0.0443
D29G129	2	30.00	15.75	12.70	10.43	0.0329
D29G181	2	26.45	17.25	10.73	10.10	0.037
D29G189	2	27.00	18.09	11.13	12.23	0.0183
D29G190	2	21.85	16.60	13.30	15.04	0.0594
D29G239	2	26.65	16.62	13.10	9.47	0.0567
D29G279	2	33.35^∗^	19.27	11.32	16.63^∗^	0.0919
D29G282	2	27.95	15.98	11.64	14.05	0.0544
D29G105	3	25.10	18.59	16.77^∗^	16.43^∗^	0.0375
D29G119	3	19.65	19.34	12.27	15.77	0.0791
D29G127	3	27.35	17.15	11.20	16.20	0.0567
D29G138	3	28.00	20.23^∗^	12.34	14.38	0.1106
D29G161	3	26.40	15.74	12.10	12.45	0.0302
D29G165	3	29.10	18.80	11.39	15.20	0.0672
D29G171	3	23.35	16.42	10.85	10.30	0.0416
D29G176	3	28.55	18.17	13.13	10.75	0.0265
D29G202	3	28.60	21.43^∗^	13.67	17.39^∗^	0.0786
D29G228	3	32.35^∗^	16.75	13.07	10.35	0.0233
D29G257	3	29.00	13.80	11.30	11.18	0.0791
D29G263	3	31.60^∗^	13.47	11.59	9.17	0.0183
D29G278	3	22.00	11.60	12.87	9.79	0.0343
D29G283	3	35.05^∗^	20.17^∗^	13.67	15.74	0.0229
D29G301	3	26.15	20.58^∗^	15.77^∗^	18.71^∗^	0.0768
D29G303	3	35.40^∗^	20.97^∗^	13.44	15.88	0.0622
Mean		28.33 ± 4.68	17.94 ± 2.53	12.84 ± 1.68	13.29 ± 2.97	0.051 ± 0.025
GPDQ3	5	25.55	15.50	11.92	10.07	
GPDQ4	5	26.20	18.37	13.17	14.30	

*^∗^Suggested significant at *p* < 0.05 calculated by *t*-test.*

### SNP Markers Generated by tGBS

In the raw SNP data, there were 12,288 polymorphic sites in F_4_ lines, parents and grandparents with minimum call rate more than 20%. We firstly removed 2,390 loci (19.4%) missing and 654 (5.3%) heterozygous in the parents. The remaining 9,244 SNPs were evenly distributed on the genome (**Table 3** and **Supplementary Figure S1a**). The 9,244 SNPs were used for constructing the parental graphical genotypes. From these SNPs, 2,188 (23.7%) were polymorphic between the parents, which were unevenly distributed across the rice genome, ranging from 57 on chromosome 12 to 371 on chromosome 1, with an average spacing of ~216.8 kb, ranging from 55.3 kb on chromosome 10 to 416.4 kb on chromosome 12 (**Table 3** and **Supplementary Figure S1b**). There were 38 large gaps, ranging from 2.0 Mb up to 6.8 Mb located on all chromosomes except chromosome 10 (**Supplementary Figure S1b**). The 2,188 SNP markers were used for QTL mapping.

**Table 3:** Distributions of SNP markers on chromosomes.

Chr	9,242 SNP			2,188 SNP		
	No.	Size (Mb)	Spacing (kb)	No.	Size (Mb)	Spacing (kb)
Chr1	999	43.0	43.1	371	43.0	116.1
Chr2	813	35.9	44.2	238	31.8	134.0
Chr3	589	36.1	61.4	91	30.6	340.2
Chr4	1182	35.2	29.8	294	35.0	119.4
Chr5	699	29.7	42.6	126	29.2	233.8
Chr6	614	31.1	50.7	95	22.2	236.0
Chr7	698	29.6	42.4	69	25.8	380.1
Chr8	752	28.3	37.6	174	28.0	161.6
Chr9	618	22.5	36.4	72	21.0	295.4
Chr10	885	22.9	25.9	346	19.1	55.3
Chr11	844	28.9	34.2	255	28.7	113.2
Chr12	551	27.4	49.8	57	23.3	416.4
Total	9,244	370.6	41.5	2,188	337.7	216.8

### Graphical Genotypes of the Parents

Using the high density SNPs, we were able to reconstruct the genome of female parent GPDQ3. It contains ~197.77 Mb (53.2%) of the WTR1 (the recipient) genome consisting of 62 chromosomal fragments with sizes ranging from ~0.37 Mb to ~14.91 Mb, and 174.19 Mb (46.8%) of the Khazar (donor 1) genome comprising 55 fragments with sizes ranging from ~0.36 Mb to ~15.22 Mb. The male GPDQ4 parental genome contains ~189.38 Mb (50.9%) of the WTR1 genome consisting of 75 chromosomal fragments with sizes ranging from ~0.32 Mb to ~12.80 Mb, and ~182.58 Mb (49.1%) of the BG300 (donor 2) genome comprising 73 chromosomal fragments with sizes ranging from ~0.44 Mb to ~18.48 Mb (**Supplementary Figure S2**).

### Identification of QTL for ST

**FIGURE 2:**
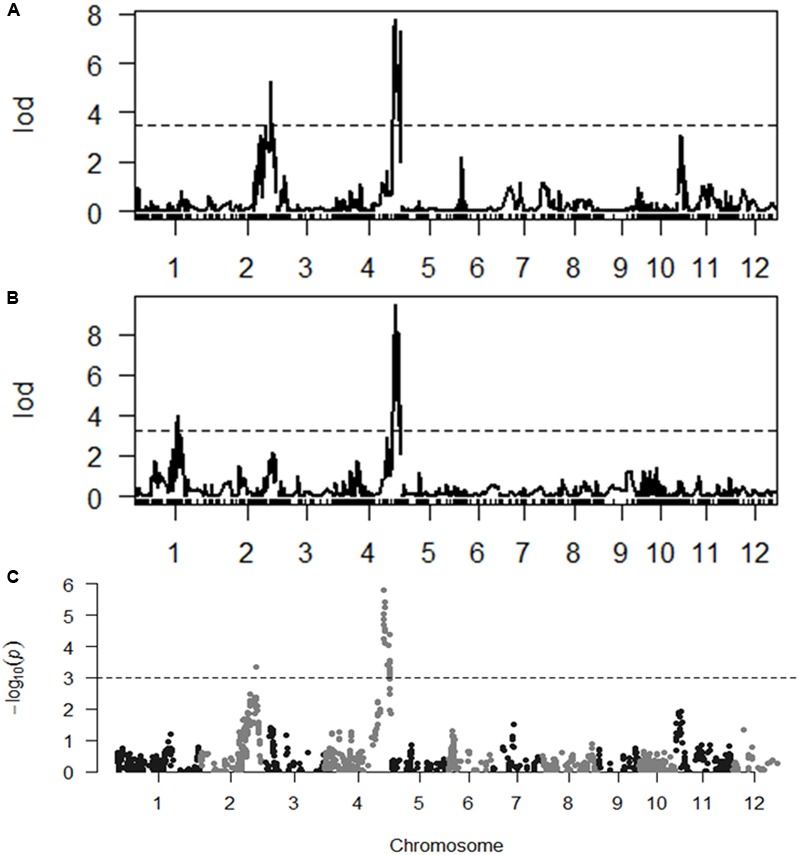
**(A,B)** The LOD curve for a genome scan of Standard Evaluation System (SES) and chlorophyll content. The horizontal dashed line represents the threshold computed by 1000 permutations. **(C)** The *p*-value of segregation distortion test for whole genome SNP markers in selected population. The horizontal dashed line indicated the *p*-value at the threshold of *q*-value being 0.05.

With the thresholds of LOD 3.5 and 3.2 obtained by the permutation for claiming QTL associated with SES and chlorophyll content, respectively, we were able to identify two QTL for each trait. *qSES2* for SES was mapped to the region of 29,420.2–29,240.3 kb on chromosome 2, explaining 12.1% of the total phenotype variance. The GPDQ3 allele could enhance tolerance to salt stress. *qSES4* and *qChlo4* for SES and chlorophyll content, respectively, were located in similar chromosome region ranging from 31,217.6 to 31,564.8 kb on chromosome 4 and their phenotype variance accounted for were 18.2% and 19.6%, respectively. The alleles from GPDQ4 at both QTL could enhance tolerance to salt stress and increase chlorophyll content. *qChlo1* for chlorophyll content was identified in the region of 23,512.5–22,210.9 kb on chromosome 1 and explained 10.4% of phenotype variance. The allele from GPDQ4 could increase chlorophyll content (**Table 4** and **Figures 2A,B**).

**Table 4:** Identified QTL for salt tolerance at seedling stage.

QTL^a^	Peak SNP position^b^	Interval (kb)	LOD	Add^c^	PVE (%)^d^	
*qSES2*	S2_29420210	29,240.3-29,544.9	5.3	0.32	12.06	
*qSES4*	S4_31217630	31,087.5-32,013.5	8.4	-0.51	18.20	
*qChlo1*	S1_23512492	22,210.9-23,525.6	4.8	2.83	10.43	
*qChlo4*	S4_31826786	31,564.8-32,159.8	9.5	3.70	19.63	

**QTL^e^**	**Peak**	**Interval (kb)**	**(A/B)_1_^f^**	**(A/B)_2_^g^**	***p***	***q***

*qSES2*	S2_29420210	29,369.5–29,544.9	0.42/0.58	0.69/0.31	0.0004	0.029
*qSES4*	S4_31826800	31,087.5–32,159.8	0.55/0.45	0.19/0.81	4.0E–06	0.004

*^a^QTL detected by linkage mapping. ^b^SNP was named by it chromosome number plus physical position. ^c^Add is additive effects associated with alleles from GPDQ4. ^d^PVE (%): the total phenotype variance explained. ^e^QTL detected by selective genotyping method. ^f^The allele frequencies of the parents, GPDQ3/GPDQ4 in the whole population. ^g^The allele frequencies of the parents, GPDQ3/GPDQ4 in the selected salt tolerant population (lines with SES from 1 to 3).*

The two QTL for SES were also detected by the selective genotyping method. For *qSES2*, its allele deviated toward GPDQ3, but for *qSES4*, its allele deviated toward GPDQ4 (**Table 4** and **Figure 2C**), indicating that the favorable alleles of *qSES2* and *qSES4* were from GPDQ3 and GPDQ4, respectively, which were consistent with the results of linkage QTL mapping.

### Candidate Gene Analysis

In the *qSES2* region, there are 2,473 SNPs in 18 genes in the Rice SNP-Seek Database, ranging from 49 SNPs for *LOC_Os02g48184* to as many as 307 SNPs for *LOC_Os02g48110*. Of the 2,473 SNPs, 299 SNPs were polymorphic among the three grandparents (WTR1, Khazar, and BG300). Most of these polymorphisms were synonymous mutations, but 61 (20.4%) SNPs were non-synonymous in 13 genes. These genes were polymorphic between parents as well and were considered as the candidate genes of *qSES2* (Supplementary Table S1).

For *qSES4* (*qChlo4*), there are 38,273 SNPs in 131 genes, ranging from 19 SNPs for *LOC_Os04g52684* to as many as 1,895 SNPs for *LOC_Os04g53160*. Among the 38,273 SNPs, only 97(6.3%) SNPs in 49 genes were non-synonymous, of which 34 genes showed polymorphism between the parents (GPDQ3 and GPDQ4) and were considered as the candidates of *qSES4* (*qChlo4*) (Supplementary Table S2).

For *qChlo1*, we found 25,271 SNPs in 124 genes in the Rice SNP-Seek Database, ranging from 8 SNPs for *LOC_Os01g40220* to as many as 1,219 SNPs for *LOC_Os01g40499*. Of the 25,271 SNPs, 1,451 were polymorphic among the three grandparents with only 103 (7.1%) non-synonymous SNPs in 40 genes, which were considered as the candidates of *qChlo1* (Supplementary Table S3).

## Discussion

### Breeding ST Varieties with Improved Yield

In this study, we demonstrated that the DQP approach was also powerful for improving target complex traits that were not originally selected in the parental ILs. We used this kind of design based on the two reasons. The first was that the parental ILs each carries a significant portion of a donor genome such that the population derived from the DQP cross would generate sufficient genetic variation for other target traits. Second, the primary target trait, ST, in this study is known to be partially correlated with the traits originally selected in the parental ILs. Apparently, both conditions were met in this study. The parental WTR1 ILs were developed by going through three rounds of strong phenotypic selection for tolerances to drought and submergence, and for GY performance under the normal conditions, and thus are tolerant to both drought and submergence. In fact, both parental WTR ILs carry nearly 50% of their donor genomes, almost twice as much as the expected 25% from their BC_1_ nature (**Supplementary Figure S2**), suggesting that the original strong phenotypic selection tended to select a much greater portion of the donor genomes. Thus, the cross between the two half-sib parental ILs was able to generate sufficient genetic diversity for the target traits such as ST and GY in this study. Although all the parents and grandparents were just moderately resistant to salt stress (SES was 5), we identified 28 lines that showed SES of 1–3 similar or even better than the tolerant check FL478. Consistent with this, the QTL mapping results indicated that favorable alleles at different ST loci were from both parents and grandparents. Thus, pyramiding the favorable alleles of different ST loci was responsible for the transgressive segregations for ST in the progeny. Similar result was obtained for GY performance under the normal irrigated conditions, suggesting the high yield alleles in the parents were non-allelic. One interesting finding was the low genome-wide heterozygosity in the F_4_ progeny in this population, which was 0.058 ± 0.034 and 0.051 ± 0.025 for the whole population and the 28 selected ST lines, or only 46% or 40% of the expected 0.125 in the F_4_ progeny. Consistent with this, almost all F_4_ lines showed tremendous phenotypic uniformity. This type of greatly reduced heterozygosity the random F_4_ population was surprising since unlike those previously reported reduced heterozygosity in backcross progenies selected under drought, salinity, and submergence (Li and Zhang, 2013; Wang et al., 2015; Ali et al., 2017). However, we found that this greatly reduced heterozygosity in the random F_2_ populations was inherited from their drought/salt selected parental lines (Li et al., 2017). Thus, we could infer that the reduced heterozygosity in the F_4_ progeny observed in this study was inherited from their drought selected parental ILs, even though the underlying epigenetic mechanism(s) remain to be elucidated. Nevertheless, using stress-selected lines as parental lines could have a major advantage to speed up the homozygosity of breeding progenies, as justified in this case by the development of six promising highly homozygous lines with significantly improved ST and yield potential in the normal and/or saline conditions from a single population in 3 years.

### Identified QTL for SES and Chlorophyll Content under Salt Stress

In this study, SES and chlorophyll content were applied to evaluation of rice ST. SES reflects the overall response of a rice plant to salt stress and was always used to evaluating rice ST (Rahman et al., 2016). Under salt stress, both osmotic stress and the accumulation of Na^+^ in leaf tissues could result in leaf fading (Munns and Tester, 2008). Thus, utilizing chlorophyll content as an indicator for ST is reasonable. Previous QTL mapping studies also identified many QTL for these two traits (Zang et al., 2008; Thomson et al., 2010; De Leon et al., 2016). With the tGBS technology (Schnable et al., 2013), we generated 12,288 SNPs with a minimum call rate > 20%. After filtering, we used 2,188 SNPs for QTL mapping. We noted 38 large gaps (>2 Mb) in the resulting linkage map due to monomorphic SNP markers shared between parents in these regions as expected, but we detected polymorphic SNP markers among grandparents in these regions (**Supplementary Figure S1**).

### Candidate Genes of Identified QTL

Utilizing the 2,188 SNPs, we were able to define identified ST QTL in narrow confidence intervals ranging from 304.6 kb for *qSES2* to 1,314.7 kb for *qChlo1* (**Table 4**). The *qSES4* and *qChlo4* were mapped to a region of 1,072.3 kb from 31,087.5 kb to 32,159.8 kb on chromosome 4 where a previously identified QTL for the two investigated traits were reportedly flanked by SSR markers RM3834 and RM127 in a ~3.0 Mb region (Thomson et al., 2010). These results indicated that increasing marker density could considerably narrow QTL intervals and shortlist the number of candidate QTL genes. Utilizing the high density SNP data of grandparents and graphical genotypes of parents, we obtained 13, 34, and 40 candidate genes for *qSES2, qSES4* (*qChlo4*), and *qChlo1*, respectively. The results demonstrated that linkage mapping using high density SNPs coupled with saturated SNPs of parents and grandparents could be a powerful strategy to shortlist candidate genes of identified QTL. Some of candidates appeared to have the same putative functions as those of known ST related genes according to their functional annotation in the MSU Rice Genome Annotation Project Database.

Among the 13 candidate genes of *qSES2, LOC_Os02g48100* was a putative DEAD-box ATP-dependent RNA helicase gene, which was same as a known ST related gene *OsDBH* that could be up-regulated in response to NaCl treatments (Macovei and Tuteja, 2012). Another candidate *LOC_Os02g48140* encoded a putative hsp20/alpha crystallin family protein, which was same as the protein product of *OsHsp17.0* (Zou et al., 2012). Heat shock proteins (Hsps) played an indispensable role in plant abiotic and biotic stress tolerances (Park and Seo, 2015). The *OsHsp17.0* over-expressed transgenic rice plants showed high tolerance to salt stress, low relative EC, low malondialdehyde content and high free proline content under salt stresses compared with the wild-type plants, which demonstrated that *OsHsp17.0* played an important function on ST (Zou et al., 2012). Therefore, the above two genes were regarded as the most likely candidates for *qSES2* (**Table 5**).

**Table 5:** The most likely candidate genes of identified QTL.

QTL	Candidate gene	Putative function
*qSES2*	*LOC_Os02g48100*	DEAD-box ATP-dependent RNA helicase, putative, expressed
	*LOC_Os02g48140*	Hsp20/alpha crystallin family protein, putative, expressed
*qSES4*(*qChlo4*)	*LOC_Os04g52560*	Transposon protein, putative, unclassified, expressed
	*LOC_Os04g52590*	Protein kinase domain containing protein, expressed
	*LOC_Os04g52810*	No apical meristem protein, putative, expressed
	*LOC_Os04g53700*	Zinc finger protein, putative, expressed
	*LOC_Os04g53740*	Thioredoxin, putative, expressed
*qChlo1*	*LOC_Os01g39890*	Transposon protein, putative, unclassified, expressed
	*LOC_Os01g39970*	Protein kinase domain containing protein, putative, expressed
	*LOC_Os01g40430*	WRKY27, expressed
	*LOC_Os01g40540*	Lectin receptor-type protein kinase, putative, expressed
	*LOC_Os01g40830*	Transposon protein, putative, mutator sub-class, expressed
	*LOC_Os01g40870*	Aldehyde dehydrogenase, putative, expressed

Among the 34 candidates of *qSES4* (*qChlo4*), *LOC_Os04g52810* encoded a putative no apical meristem protein that was a NAC domain transcription factor playing important roles in regulating osmotic stress tolerance in plants (García-Morales et al., 2014). Many NAC domain transcription factors have been demonstrated to have significant role in ST of rice. For instance, *SNAC2, ONAC045*, and *OsNAC5* were induced by high salt and their over-expression could enhance rice plants tolerance to salt stress (Hu et al., 2008; Zheng et al., 2009; Takasaki et al., 2010). García-Morales et al. (2014) had reported that different expression patterns of two NAC genes (*NAC* and *NAC32*) could result in better performance of Cotaxtla plants compared with TresRíos plants under salt stress. Another gene *LOC_Os04g52590* encoded a putative protein kinase domain containing protein that was same as the encoded protein of a known ST related gene *DSM1* (Mitogen-activated protein kinase gene). The expression of *DSM1* was induced by salt and the *dsm1* mutant rice plant was more sensitive to salt stress (Ning et al., 2010). The candidate *LOC_Os04g53740* is a thioredoxin gene, which was same as *OsTRXh1* whose over-expression would lead to salt-sensitive phenotype (Zhang et al., 2011). The candidate gene *LOC_Os04g52560* encoded a putative transposon protein. Two known ST related genes *OsAOX1a* and *OsAOX1b* also encoded transposon protein and their expression was induced by salt treatment (Ohtsu et al., 2002). The gene *LOC_Os04g53700* encoded a putative zinc finger protein. Many ST related genes encoding zinc finger protein have been identified. For instance, *OsSRZ1* encoded a protein with three C2C2-type zinc finger motifs and its expression was markedly repressed by salt (Huang et al., 2008); *OsiSAP8* encoded a zinc finger A20 and AN1 domain-containing stress-associated protein and its over-expression enhanced the tolerance of rice plant to salt stress (Kanneganti and Gupta, 2008); *OsDSG1* encodes a zinc finger C3HC4 type domain containing protein having E3 ubiquitin ligase activity and its mutant rice plant had greater tolerance to high salt stress (Park et al., 2010). Therefore, the above five genes were treated as the most likely candidates for *qSES4* (*qChlo4*) (**Table 5**).

Among the 40 candidate genes of *qChlo1, LOC_Os01g39890* and *LOC_Os01g40830* were transposon protein genes, which were same as *LOC_Os04g52560*, one of the candidate genes of *qSES4* (*qChlo4*) discussed above. *LOC_Os01g39970* had same gene produce with one of the candidate genes of *qSES4* (*qChlo4*), *LOC_Os04g52590* as discussed above. Another gene *LOC_Os01g40430* encoded WRKY27, a WRKY transcription factor. Previous studies have found that *WRKY13* and *WRKY45* could negatively regulate rice response to salt stress (Tao et al., 2011; Xiao et al., 2013). The candidate *LOC_Os01g40540* encoded a putative lectin receptor-type protein kinase that was same as the encoded protein of *SIT1*. Li et al. (2014) reported that the *SIT1* mediated ethylene production and salt-induced ethylene signaling and its RNAi transgenic rice plant showed enhanced ST. The candidate *LOC_Os01g40870* was a putative aldehyde dehydrogenase gene, which was same as *OsBADH1*. RNAi-directed down-regulation of *OsBADH1* could result in decreased tolerance to salt stress, which was mainly attributed to the decline of ability to dehydrogenate the accumulating metabolism-derived aldehydes (Tang et al., 2014). Thus, the above six genes were proposed to be the most likely candidates of *qChlo1* (**Table 5**).

Although many candidate genes were identified, the results were just based on a single reference genome of *Geng* (*japonica*) species. As both parents in the current studied population were belonging to *Xian* (*indica*), it’s possible that some specific genes in *Xian* might be missing in the Nipponbare reference genome. Thus, as more and more *Xian* reference genomes become available, such as 93-11, MH63 and ZS97, SNPs are being recalled according to these *Xian* reference genomes utilizing our raw tGBS data of the studied population to identify candidate genes.

## Conclusion

Using a DQP population derived from a cross between two selected ILs, we developed six promising and largely homozygous lines with significantly improved ST and yield potential in normal and/or saline conditions in 3 years. We also demonstrated that the tGBS was an efficient technology to generate high density SNPs for closed related parents for QTL mapping and to shortlist candidate genes for three ST QTL *qSES2, qSES4* (*qChlo4*), and *qChlo1* by taking advantage of the SNP data from 3K RGP. Functional analyses of the candidate genes for the ST QTL allowed us to infer two, five, and six genes as the most likely candidates of *qSES2, qSES4* (*qChlo4*), and *qChlo1*, respectively. These candidate genes of new loci for rice ST provide valuable information for future functional characterization and marker-assisted selection based breeding for improving rice tolerance to salt stress.

## Author Contributions

ZKL, JA, and JX designed the experiment; YP and XW performed the phenotypic collection; YP, XW, and WW performed the analysis and interpretation of the data; ZKL, YP, KC, and JA drafted the paper; all authors revised the paper and approved the final version to be published.

## Conflict of Interest Statement

The authors declare that the research was conducted in the absence of any commercial or financial relationships that could be construed as a potential conflict of interest.

## Footnotes

1 http://snp-seek.irri.org/index.zul

2 http://bbi.irri.org

3 http://www.qiagen.com

4 http://rice.plantbiology.msu.edu/

## Supplementary Material

FIGURE S1 The physical maps of the 9,242 SNP data set **(a)** and the 2,188 SNP data set **(b)**.

FIGURE S2 The graphical genotypes of GPDQ3 and GPDQ4. B, K, and W indicated the genotype from BG300, Khazar, and WTR1, respectively.
