# Supplementary figures and images for: Simultaneous Improvement and Genetic Dissection of Salt Tolerance of Rice (Oryza sativa L.) by Designed QTL Pyramiding
Source: Front Plant Sci. 2017 Jul 20;8:1275. doi: 10.3389/fpls.2017.01275 (PMC5517400; doi:10.3389/fpls.2017.01275)

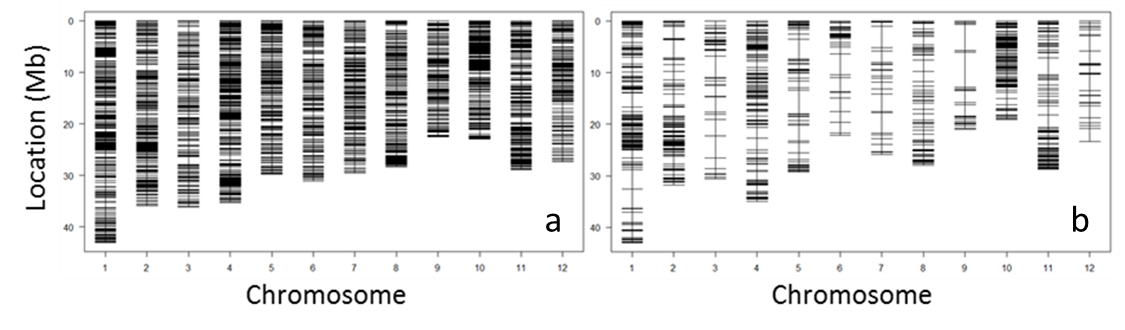

Supplement: FIGURE S1 — The physical maps of the 9,242 SNP data set (a) and the 2,188 SNP data set (b). [file Image_1.TIF]

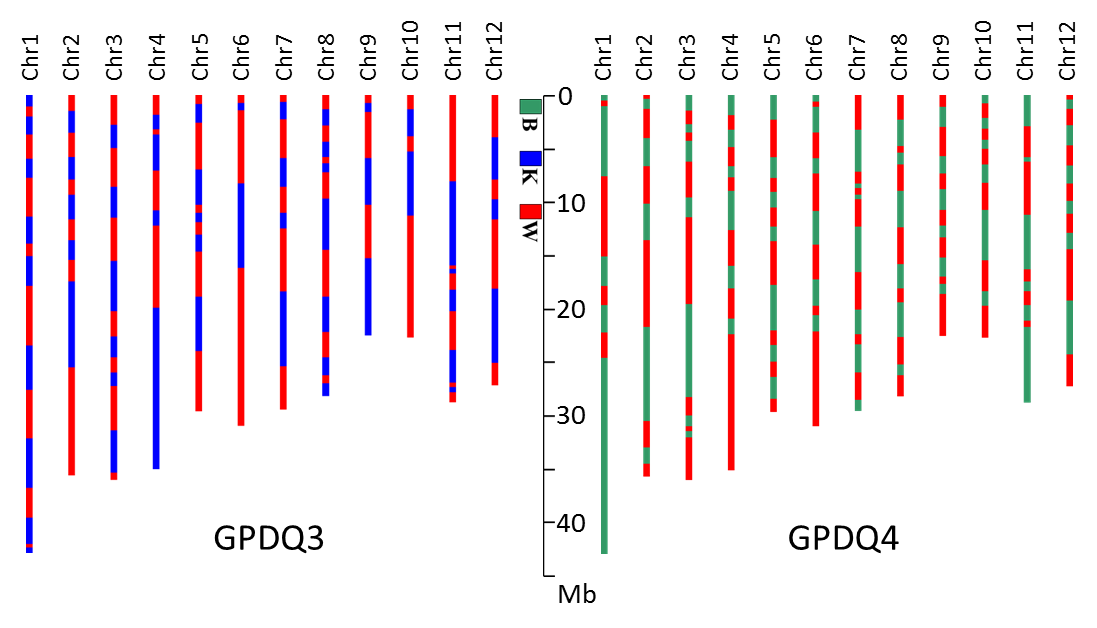

Supplement: FIGURE S2 — The graphical genotypes of GPDQ3 and GPDQ4. B, K, and W indicated the genotype from BG300, Khazar, and WTR1, respectively. [file Image_2.TIF]
